# An archaeo-metabolomics approach for identifying cedar tar in archaeological samples: differentiating plant products and production processes

**DOI:** 10.1038/s41598-026-50080-6

**Published:** 2026-05-05

**Authors:** Barbara Huber, Océane Pollet, Sahar B. Kandil, Dennis Graen, Lou Spanneut, Daniel Giddings Vassão, Lindsay Mas-Normand, Céline Joliot, Elizabeth Roche, Sylvain Burri, Aline Durand, Martine Regert, Ricardo Fernandes, John A. Pickett, Gérald Culioli, Thibaut Devièse

**Affiliations:** 1 Institute of Archaeological Science, University of Tübingen, Tübingen, Germany; 2 Archaeometry Research Group, Institute for Pre- and Protohistory and Medieval Archaeology, University of Tübingen, Tübingen, Germany; 3 Department of Coevolution of Land Use and Urbanisation, Max Planck Institute of Geoanthropology, Jena, Germany; 4 Centre de Recherche et d’Enseignement en Géosciences de l’Environnement (CEREGE), CNRS, IRD, INRAE, Collège de France, Aix-Marseille Université, Aix-en-Provence, France; 5 Physique des Interactions Ioniques et Moléculaires (PIIM), CNRS, Aix-Marseille Université, Marseille, France; 6 School of Chemistry, Cardiff University, Cardiff, UK; 7 Institute of Ancient Classics (Institut für Alterumswissenschaften), Friedrich-Schiller-Universität Jena, Jena, Germany; 8 CNRS, Minist Culture, LAMPEA, UMR 7269, Aix Marseille University, Aix-en-Provence, France; 9 Department of Biochemistry, Max Planck Institute for Chemical Ecology, Jena, Germany; 10 Laboratory and Fieldwork Unit, Max Planck Institute of Geoanthropology, Jena, Germany; 11 IMBE, CNRS, IRD, Aix Marseille Université, Avignon Université, Avignon, France; 12 TRACES UMR 5608 CNRS, Université Toulouse Jean Jaurès, Toulouse, France; 13 Department of History, CReAAH-UMR 6566, Le Mans Université, Le Mans, France; 14 CNRS, CEPAM, Université Côte d’Azur, Nice, France; 15 Department of Archaeology, Max Planck Institute of Geoanthropology, Jena, Germany; 16 School of Archaeology, Faculty of Archaeology, University of Warsaw, Warsaw, Poland; 17 Climate Change and History Research Initiative, Princeton University, Princeton, NJ USA

**Keywords:** Biochemistry, Chemistry, Plant sciences

## Abstract

The human ability to create manufactured substances is not a modern innovation. It began in the Middle Palaeolithic with the production of birch tar through dry distillation, a process of heating bark under low-oxygen conditions. Over time, this technological knowledge extended to other materials and purposes. Among these, cedar products became especially significant, valued in medicine, technology, cosmetics, and mummification. Yet, distinguishing cedar tar from other cedar products, such as resin or essential oil, has long challenged archaeological science due to their closely related chemical composition. Here we used a GC–MS-based metabolomics approach to identify chemical markers of cedar tar samples. Using modern *Cedrus atlantica* samples, we defined a set of diagnostic compounds (i.e. dihydro-ar-turmerone, cuparene, dihydrocurcumene, ar-himachalene and 1-methyl-3,5-diisopropylbenzene) that reveal both the cedar origin and the transformative process of dry distillation. As a proof of concept, these chemical markers were searched for and successfully detected in ancient Egyptian mummification balms (664–332 BCE), providing direct evidence for cedar tar use in embalming mixtures and new insights into ancient technologies and cultural practices.

**Supplementary Information:**

The online version contains supplementary material available at 10.1038/s41598-026-50080-6.

## Introduction

The human capacity to produce manufactured substances from plant materials dates back tens of thousands of years, demonstrating a profound understanding of natural resources and transformative processes since prehistoric times^1^. Evidence suggests that as early as the Middle Palaeolithic (approx. 200,000 years), Neanderthals demonstrated an advanced level of technological ingenuity by altering natural materials, such as bark, to produce manufactured materials, such as birch tar^2,3^. Unlike resins, which are natural exudates that may form hardened deposits on trees^4^, tars must be produced through deliberate transformative processes^5,6^. These involve controlled heating to obtain tar from raw plant materials, such as the bark of birch trees (*Betula* spp.), or the wood of coniferous trees including cedars (*Cedrus* spp.), junipers (*Juniperus* spp.) or pines (*Pinus* spp.)^7,8^. Tar is produced by dry distillation, a pyrolytic process, in which wood is heated in a low-oxygen environment^9^. Under these conditions, thermal decomposition releases volatile compounds, comprising both native plant metabolites and thermally derived products. As the vapours cool, heavier organic compounds condense to form tar, a dense viscous material rich in aromatic hydrocarbons and sesquiterpenoids^6^, and, depending on the wood taxon, also di- or triterpenoids^10^.

Previous analyses of archaeological tars have primarily focused on birch bark tar and pine tar^3,5,9,11^. Among these, birch bark tar has received particular attention due to its early use in prehistory, including by Neanderthals^2,12–15^. Conifer-derived tars, particularly pine tar, have also been studied, given their versatility and broad applications in ancient times. Pine tar has been commonly used as a waterproofing agent for vessels and shipbuilding, as an adhesive, and even as an ingredient in medicinal preparations^8,11,16–19^. In contrast, cedar tar has received little attention, despite its historical significance and claimed bioactive properties. In ancient Egypt, for example, cedar products are known to have played a crucial role in the mummification process and were widely used in perfumery and cosmetics^20–22^. However, differentiating between cedar-derived substances, particularly between tars and essential oils, remains challenging. Since all these plant materials—resins, tars, and oils—originate from the same organism, they often exhibit significant overlaps in their molecular composition^6,7,23^. The presence of similar plant specialised metabolites poses a challenge for distinguishing between these plant materials, especially in archaeological contexts, where natural degradation processes may have further altered their original chemical profiles^22^. Previous studies have encountered challenges in distinguishing between plant materials coming from the same organism, highlighting the need for more precise analytical developments^22,24^.

The chemical composition of modern cedar-derived materials has been investigated in several studies. In particular, essential oils of *Cedrus* species have been extensively analysed, largely due to their reported bioactive properties and biological potential^25–27^. Cedar-derived materials are characterised by sesquiterpenes, such as himachalenes and atlantones, which are recognised as taxonomic markers of cedar^6,28,29^. Himachalenes are a group of bicyclic sesquiterpenoids characterised by a fused-ring skeleton composed of one six-membered ring and one seven-membered ring. They are biosynthetically derived from farnesyl pyrophosphate (FPP) through cyclization reactions catalysed by sesquiterpene synthases^28,30^. The three principal isomers detected in cedar are α-, β-, and γ-himachalenes (Fig. S3). In addition, related oxygenated, unsaturated, and aromatised derivatives such as dehydrohimachalenes, himachalols, and ar-himachalenes are also present in cedar products. Another prominent class of diagnostic compounds are atlantones, a group of sesquiterpene ketones biosynthetically derived from the same bicyclic himachalane skeleton^28^. These compounds—including *cis*- and *trans*-α- and γ-atlantones (Fig. S3)—are prominent constituents of *Cedrus* spp. essential oils, occurring alongside himachalenes^29,31^. Atlantones and their derivatives are also detected in cedar tars but are largely absent from exuded resins, which are instead dominated by monoterpenes and diterpenoids, particularly of the abietane and pimarane types^7,32–34^.

Himachalenes have been reported to occur almost exclusively in cedar^26,33,35^, although certain himachalene isomers have also been detected in *Juniperus* species (Cupressaceae family). For example, β-himachalene is present, but only in trace amounts, in *Juniperus communis*^36^ and *Juniperus phoenicea*^37^. Moreover, chemical studies on *Juniperus oxycedrus* have not detected himachalenes^35,38^, further supporting their strong diagnostic value for cedar materials. A comparable pattern has been observed in *Abies* species. While some studies have reported the occurrence of some himachalene isomers in low concentrations^39^, other investigations have not detected them^40^. This likely reflects interspecific variations and the trace-level abundance of these compounds in some specific taxa, which may fall below detection thresholds depending on the analytical method employed.

Himachalenes (particularly ar-himachalenes) have previously been proposed as diagnostic chemical markers for identifying cedar products in archaeological residues^41^, and have been successfully detected in several archaeological contexts^21,22,42^. However, while these compounds enable taxonomic attribution to *Cedrus*, they do not allow differentiation between distinct cedar-derived products. In previous studies, the presence of himachalenes has therefore generally been interpreted only as evidence for cedar in a broad sense. For example, residues from an embalming substance recovered from a mummified skull yielded himachalenes and were interpreted as indicating the presence of “a natural resin, pitch, or tar”^42^. Similarly, organic residues recovered from ancient Egyptian vessels contained himachalenes but were broadly assigned to “cedar oil or tar”^22^. These examples illustrate that although cedar-derived materials can be identified, their corresponding specific product type often remains unresolved. The ability to distinguish between different cedar products is nevertheless important, as these substances result from different production processes and therefore reflect distinct technological practices.

Recent advances in mass spectrometry-based metabolomics have provided a comprehensive approach to distinguishing such complex archaeo-organic materials^43,44^. This type of analytical approach involves the systematic profiling of metabolites, i.e., low-molecular weight molecules (< 1500 Da) that function as intermediates or end products of metabolic pathways^45^. Because metabolite profiles can be species- and tissue-specific, metabolomics has proven particularly useful for distinguishing closely related plant taxa through the identification of taxonomic chemomarkers (chemical markers)^43,46–48^. Beyond taxonomic discrimination, metabolomics can also be used to identify chemomarkers associated with specific processing pathways. Thermal or technological transformations, such as heating or distillation (e.g. in the production of tars or essential oils), can alter the original metabolite composition and generate new diagnostic compounds that reflect particular production processes^10,41,47,49^. Metabolites can therefore serve as indicators of specific natural materials and processing methods. This makes metabolomics particularly valuable for identifying thermally altered products such as tars, which typically no longer contain recoverable DNA or proteins^50^, but still retain diagnostic metabolites^51,52^.

Here, we present a GC–MS-based metabolomics approach to identify chemomarkers that distinguish cedar tar from other cedar-derived products. Applied to traditionally produced tar samples, as well as resins, and essential oils from reference plant materials (modern *Cedrus atlantica*), this method enables the identification of diagnostic chemomarkers specific to cedar tar and its production process. As a proof of concept, we further demonstrate the applicability of this approach to archaeological materials by detecting these chemomarkers in ancient Egyptian embalming substances. In addition, we show that, retrospectively applied to previously published datasets, this analytical approach enables the re-evaluation of legacy data in light of newly defined chemomarkers.

## Results

### Chemical characterisation of cedar tar

**Fig. 1:**
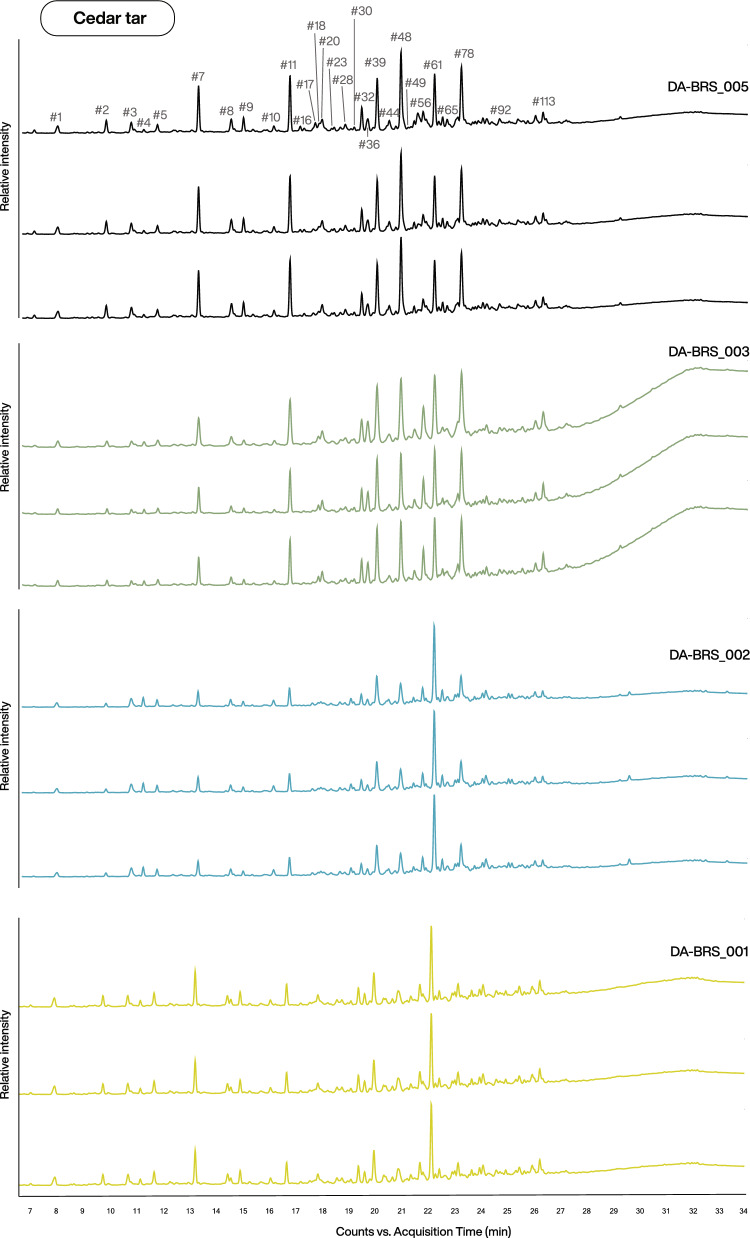
GC–MS total ion chromatograms (TIC) of modern *C. atlantica* tar samples (DA-BRS_001–005). Samples (shown in different colours) were analysed in triplicates. Feature numbers (#) annotated in the first chromatogram correspond to the compounds listed in Table 1.

GC–MS analysis of modern traditionally produced *C. atlantica* tar samples (DA-BRS_001–005, see Table S1) revealed highly comparable chromatographic profiles across all samples. Triplicates of each tar sample displayed near-identical peak patterns and relative intensity distributions, suggesting strong analytical reproducibility (Fig. 1). Although variations in peak intensities were observed between different tar samples, the overall chromatographic fingerprint remained similar across samples. All samples were characterised by a moderate number of lower-intensity peaks in the early retention-time region (between 8 and 17 min), followed by a dense cluster of high-intensity peaks between approx. 19 and 23 min. Samples DA-BRS_003 and 005 exhibited a comparatively more complex profile, with several prominent peaks of similar intensity within 20–23 min.

The central chromatographic region (17–23 min) contained the dominant sesquiterpenoid constituents of cedar tar and represented the core of the molecular fingerprint of the analysed samples (see Table 1 for compound identification). The chromatograms showed several closely eluting bicyclic and tricyclic sesquiterpene hydrocarbons. Among the identified compounds were α-cedrene (ID n° #17) and himachala-2,4-diene (#18), which represented characteristic cedrene- and himachalene-type sesquiterpenes. In addition, a longifolene-type sesquiterpene (#16) and related rearranged derivatives, such as dihydrocurcumene (#20), were detected within the same retention-time window. Alongside these hydrocarbon sesquiterpenes, several oxygenated and aromatic sesquiterpene derivatives were consistently observed. These included ar-himachalene (#39), cuparene (#32), calamenene and calamenene-type compounds (#36 and #44), dihydro-ar-turmerone (#48), and oxygenated sesquiterpenes (#30 and #92). These compounds exhibited varying degrees of dehydrogenation and rearrangement. The chromatographic profiles of samples DA-BRS_003–005 were dominated by dihydro-ar-turmerone (#48) and ar-himachalene (#39), whereas these peaks were comparatively smaller in samples DA-BRS_001–002.

**Table 1:** Compounds detected in modern *C. atlantica* tar samples.

Feature ID	Retention time (min)	Putative annotation name	Major fragment ions (*m/z*)	M⁺·
#1	7.96	3-Methyl-2-butenoic acid	**100**, 82, 83, 85, 55, 54, 41, 53, 45, 43	100
#2	9.77	4-Methyl-3-pentenoic acid	**99**, 43, 55, 70, 56, 59, 71, 41, 42, 39	114
#3	10.72	4-Oxopentanoic acid	**43**, 55, 55, 45, 73, 42, 101, 116, 44	116
#4	11.18	Cresol isomer (*m*/*p*/*o*)	**107**, 108, 77, 79, 51, 53, 90, 80, 78, 50	107
#5	11.70	2,6-Dimethyl-2-hepten-4-one	**85**, 43, 41, 56, 125, 140, 69, 55, 42, 40	140
#7	13.25	1-(4-Methylphenyl)ethanone	**119**, 91,134,65, 120, 89, 63, 43, 92, 51	134
#8	14.49	4-Methylbenzoic acid	**91**, 136, 119, 65, 92, 63, 89, 43, 90, 137	136
#9	14.97	*p*-Cymen-7-ol	**135**, 150, 105, 79, 43, 119, 77, 41, 55, 91	150
#10	16.09	Methyl 6-oxoheptanoate	**43**, 55, 58, 69, 41, 126, 88, 111, 59, 84	158
#11	16.71	1-Methyl-3,5-diisopropylbenzene	**119**, 161, 133, 105, 176, 43, 91, 115, 65, 77	176
#16	17.56	Longifolene	**161**, 94, 91, 105, 93, 107, 189, 95, 119, 140	204
#17	17.66	*α*-Cedrene	**119**, 93, 105, 91, 204, 161, 120, 41, 121, 77	204
#18	17.81	Himachala-2,4-diene	**133**, 119, 105, 204, 93, 91, 161, 69, 41, 69	204
#20	17.92	Dihydrocurcumene	**119**, 120, 204, 105, 91, 117, 118, 41, 77, 115	204
#23	17.98	Trimethyl-substituted indanone	**159**, 174, 115, 160, 91, 133, 116, 43, 128, 129	174
#28	19.01	*α*,*β*-Dimethylcinnamic acid	**176**, 115, 91, 175, 158, 130, 129, 116, 130, 161	176
#30	19.13	Oxygenated sesquiterpene	**137**, 43, 138, 95, 109, 207, 93, 41, 123, 121	222
#32	19.41	Cuparene	**132**, 131, 145, 119, 202, 105, 133, 117, 91, 115	202
#36	19.64	Calamenene	**159**, 160, 128, 129, 202, 131, 144, 115, 105, 143	202
#39	19.99	Ar-himachalene	**187**, 145, 131, 202, 128, 132, 129, 105, 115, 159	202
#44	20.45	Calamenene-type compound	**173**, 158, 143, 128, 43, 115, 159, 133, 157, 160	216
#48	20.89	Dihydro-ar-turmerone	**119**, 218, 203, 57, 161, 91, 105, 117, 118, 120	218
#49	20.93	Naphthalenone-type compound	**173**, 145, 188, 115, 91, 128, 117, 129, 174, 146	188
#56	21.72	Benzene derivative	**133**, 105, 91, 134, 43, 135, 79, 93, 41, 117	281
#61	22.16	Unidentified compound	**107**, 43, 69, 79, 41, 55, 82, 93, 196, 135	
#65	22.47	Naphthalene-type compound	**159**, 145, 131, 144, 115, 91, 202, 128, 133, 187	202
#78	23.16	3,3,4-Trimethyl-4-(4-methylphenyl)cyclopentanone	**132**, 117, 216, 133, 115, 91, 131, 116, 92, 119	216
#92	24.10	Oxygenated sesquiterpene	**127**, 82, 55, 220, 43, 41, 128, 81, 121, 111	220
#113	26.26	Alkylated naphthalene derivative	**109**, 195, 43, 82, 112, 124, 83, 41, 135, 67	224

Feature IDs (#) correspond to the labelled peaks in the chromatograms shown in Fig. 1 and to the feature identifiers used in the metabolomics data matrix. The base peak is indicated in bold, and fragment ions are listed in order of decreasing relative intensity.

In addition to the sesquiterpenoid fraction, the chromatograms of cedar tar samples revealed the presence of several low-molecular-weight aromatic and oxygenated compounds, including alkylated benzene derivatives, such as 1-methyl-3,5-diisopropylbenzene (#11), a cresol isomer (#4), and aromatic ketones, such as 1-(4-methylphenyl)ethanone (#7). Further aromatic compounds including benzoic acid derivatives (#8), and alkylated cinnamic acid derivatives (#28) were also observed. The chromatograms also contained several condensed aromatic compounds tentatively assigned as naphthalene-type derivatives (#49, #65, and #113), together with a substituted cyclopentanone derivative (#78).

The early chromatographic region was characterised by several short-chain carboxylic acids and oxygenated degradation products, including 3-methyl-2-butenoic acid (#1), 4-methyl-3-pentenoic acid (#2), and 4-oxopentanoic acid (#3). Additional minor compounds included 2,6-dimethyl-2-hepten-4-one (#5), *p*-cymen-7-ol (#9), and methyl 6-oxoheptanoate (#10). Such chemical constituents are commonly associated with the thermal degradation of lignocellulosic biomass and their occurrence is consistent with the high-temperature transformation processes involved in tar production^53^. Among these compounds, 1-methyl-3,5-diisopropylbenzene (#11) and 1-(4-methylphenyl)ethanone (#7) consistently represented the most intense peaks within this specific chemical fraction.

### Chemomarkers distinguishing cedar tar from other cedar-derived products

**Fig. 2:**
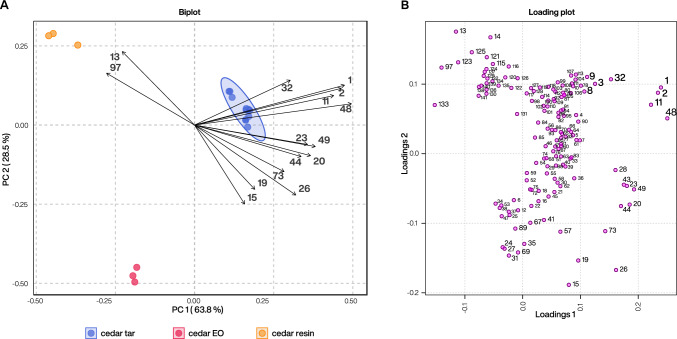
Principal component analysis (PCA) obtained from GC–MS data of *C. atlantica* tar samples compared to resin and essential oil reference samples and features contribution to sample clusterization. (**A**) PCA biplot showing the distribution of samples and the most contributing features along the first two principal components. The numbers next to the arrows represent top 15 features that contribute most to sample separation. Direction and length of arrows indicating their contribution to sample separation. (**B**) Loading plot illustrating the distribution of all features present in the samples. Features with strong positive loadings on PC1 are primarily associated with cedar tar samples, whereas features with negative PC1 loadings contribute to the separation of resin (upper part) and essential oil samples (lower part).

To identify which compounds detected in cedar tar samples represent distinctive chemomarkers for this product category, we systematically compared tar samples with reference samples of *C. atlantica* essential oil and resin using an untargeted metabolomics data-processing workflow which allowed for the generation of a final data matrix containing a total of 141 features. This data matrix was first analysed using principal component analysis (PCA) to assess the global clustering structure derived from the chemical profiles of the samples, with the aim of determining the overall separation among all samples and identifying hypothetical outliers. The resulting PCA biplot revealed a clear separation of all analysed cedar products along the first two PC axes (accounting for more than 90% of the total variance; Fig. 2A) and showed the orientation of contributing variables (i.e. features) within the same coordinate space. Cedar tar samples clustered tightly at positive PC1 values with minimal dispersion along PC2. Cedar resin samples were positioned at negative PC1 and higher PC2 values, whereas cedar essential oil samples also occupied negative PC1 but they were separated from resin samples along PC2, forming a distinct group in the lower quadrant. The vectors shown in the biplot represented the top contributing features (i.e. individual metabolites based on loading magnitude) that drove separation along the first two PCs. Several of these features were oriented toward the positive PC1 region, coinciding with the cedar tar cluster, particularly features #1, #2, #11, #32, and #48. The directionality of these vectors indicated a strong positive association with cedar tar samples relative to resin and essential oil ones.

The corresponding loading plot (Fig. 2B) displayed all feature loadings of cedar tar, resin and essential oil samples for PC1 and PC2. Several features exhibited high positive loadings on PC1, positioning them on the right side of the plot in alignment with the cedar tar cluster. In contrast, features with strongly negative PC1 loadings were located on the left side of the loading space and corresponded to the region occupied by resin and essential oil samples. Only a limited number of features displayed extreme negative PC1 values compared to the larger group extending toward positive PC1. Along PC2, features were distributed across both positive and negative values. A small number of variables occupied peripheral positions at the outer edges of the loading space, reflecting comparatively high absolute loading values, whereas the majority of features clustered closer to the origin, indicating more moderate contributions to the first two principal components.

**Fig. 3:**
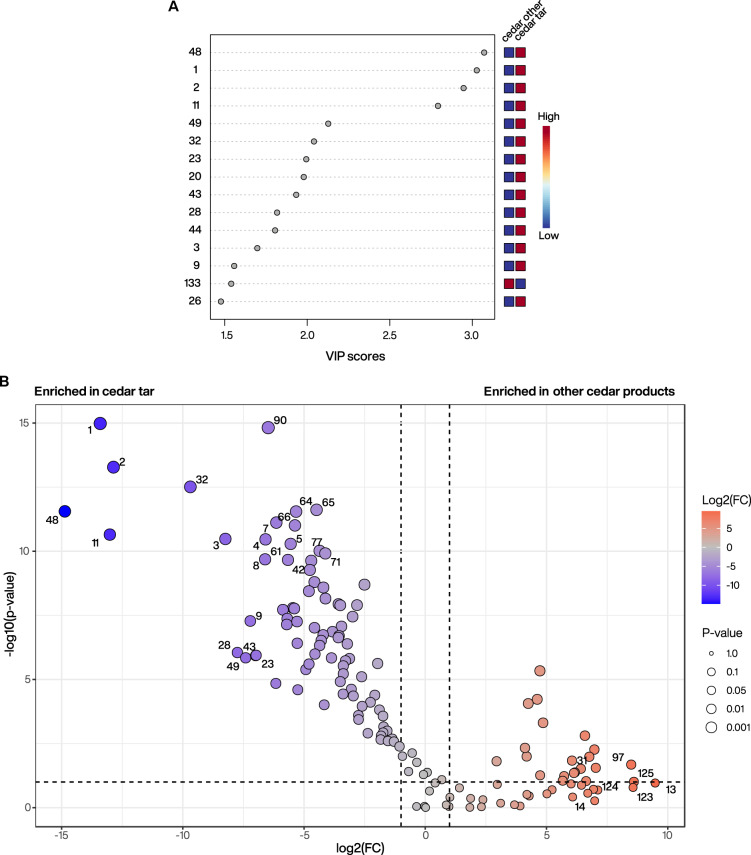
Identifying discriminant markers of cedar tar compared to other cedar products. (**A**) Variable importance in projection (VIP) plot from PLS-DA comparing cedar tar samples to essential oil and resin samples (labelled “cedar other”). The numbers on the left side correspond to individual features considered as significant (with VIP scores along PC1 > 1.0). The associated colour code illustrates relative abundance (blue = low/absent, red = high) across groups. (**B**) Volcano plot highlighting discriminant markers between cedar tar and other cedar products. Features on the left side are enriched in cedar tar samples, whereas features on the right side are enriched in other cedar products. The colour scale represents the log_2_ fold-change and the y-axis shows − log_10_ (*p* value). Points highlighted and labelled correspond to the top significant features based on fold-change and *p* values.

We then performed a partial least squares discriminant analysis (PLS-DA), a supervised multivariate approach that maximizes separation between predefined groups and highlights the most discriminating variables based on variable importance in projection (VIP) scores. The two groups we compared were cedar tar samples versus all other cedar products (collectively labelled “cedar other”; Fig. 3A). This model was designed to identify key features that distinguish cedar tar from other cedar products that are not produced by dry distillation. The PLS-DA model identified the 15 most discriminant features that drove separation between these two groups. All discriminating features of the cedar tar group identified in this model were either exclusive to the tar samples or present at only minimal concentrations in the other cedar products, highlighting their diagnostic potential for substances derived through dry distillation. Among the most significant markers were dihydro-ar-turmerone (#48), 3-methyl-2-butenoic acid (#1), 4-methyl-3-pentenoic acid (#2) and 1-methyl-3,5-diisopropylbenzene (#11), all exhibiting VIP scores (along PC1) above 2.5. Additional highly discriminating compounds included cuparene (#32) and dihydrocurcumene (#20). Consistent results were obtained in the accompanying volcano plot (Fig. 3B), which also highlighted the same key compounds as significantly enriched in cedar tar samples versus other cedar products.

Although these compounds represent characteristic features distinguishing cedar tar from other cedar-derived products, several of the identified chemomarkers also occur naturally in other plant taxa. For instance, dihydro-ar-turmerone (#48) in cedar likely originates from α-atlantone through an aromatisation process (Fig. S4) that can proceed from both the (*E*)- and (*Z*)-α-atlantone isomers, as demonstrated in previous synthetic studies^54^. Beyond this formation pathway in cedar, dihydro-ar-turmerone is also known as a reduced derivative of ar-turmerone, a prominent sesquiterpenoid abundant in the rhizomes of *Curcuma* species, particularly *Curcuma longa* (turmeric)^55–57^. Likewise, cuparene (#32) has been reported in other plant species, particularly in junipers (Cupressaceae)^20,27,36,58^. Given their broader natural occurrence, the individual detection of these chemomarkers is not sufficient to confirm the presence of cedar tar. In addition, low-molecular-weight compounds, such as #1–#3 represent general thermal degradation products, which are formed during fragmentation of larger organic precursors and therefore they can serve primarily as indicators of thermal processing rather than as taxonomic markers. However, the co-occurrence of these compounds with cedar-specific metabolites, namely aromatic himachalenes and/or atlantones, provides a diagnostic molecular signal for cedar tar.

**Fig. 4:**
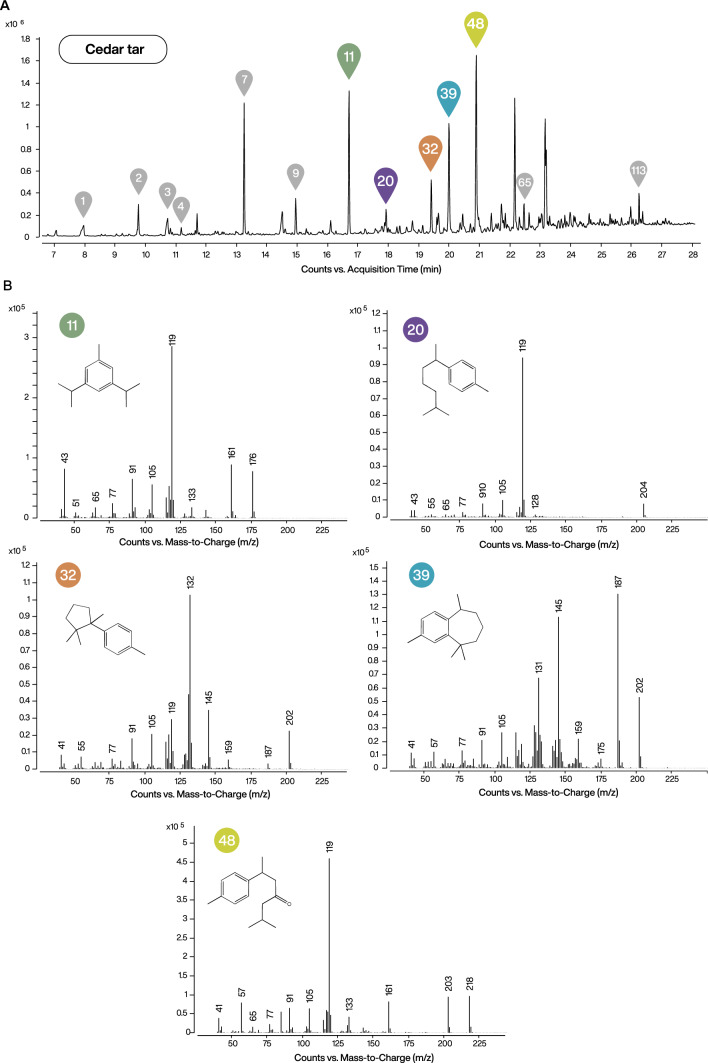
Characteristic set of chemomarkers for cedar tar samples. (**A**) Total ion current (TIC) GC–MS chromatogram of cedar tar sample DA-BRS_005 with chemomarkers indicated. Colored markers denote the primary cedar tar chemomarkers identified in this study, whereas grey markers represent additional compounds that support the identification of cedar tar when considered together with the primary marker set but are not individually diagnostic. (**B**) Mass spectra (EI, 70 eV) of the primary cedar tar chemomarkers (features #11, #20, #32, #39, and #48), corresponding to 1-methyl-3,5-diisopropylbenzene, dihydrocurcumene, cuparene, ar-himachalene, and dihydro-ar-turmerone, respectively.

Based on the statistical analysis and chromatographic observations, we proposed a characteristic set of highly discriminant chemomarkers for cedar tar (Fig. 4). These include dihydro-ar-turmerone (#48), 1-methyl-3,5-diisopropylbenzene (#11), cuparene (#32) and dihydrocurcumene (#20). Although ar-himachalene (#39) was not among the most discriminant features in the statistical model, as it is also present in other *Cedrus* products, it represents one of the most abundant compounds in the tar samples and belongs to the himachalene structural family typical for cedar-derived substances. It can therefore be included as an additional component of the cedar tar chemomarker set. In addition to these primary markers, several additional compounds consistently detected in the tar samples may support the identification of cedar tar when considered together with this marker set, although they are not individually diagnostic. These included short-chain degradation products such as 3-methyl-2-butenoic acid (#1), 4-methyl-3-pentenoic acid (#2), and 4-oxopentanoic acid (#3), as well as aromatic and oxygenated compounds such as cresols (#4), 1-(4-methylphenyl)ethanone (#7), 4-methylbenzoic acid (#8), and *p*-cymen-7-ol (#9), and the aromatic sesquiterpene calamenene (#36). The set further includes condensed aromatic compounds, including naphthalene-type derivatives (#49, #65, and #113).

Following the identification of this set of compounds characteristic of cedar tar, it is important to consider their formation pathways and preservation potential, particularly with regard to their application in archaeological contexts. These compounds are unlikely to form through natural degradation processes. Archaeological burial environments are typically characterised by low temperatures, slow oxidation, and microbial activity, conditions that promote molecular breakdown rather than the thermal rearrangements and aromatisation reactions required to generate these compounds^41,59^. In contrast, dry distillation involves elevated temperatures (typically above 250 °C) and oxygen-limited conditions that fundamentally differ from burial environments, making the in situ formation of such aromatic compounds under archaeological conditions unlikely^9^. Compounds formed through dry distillation often feature condensed aromatic ring structures and reduced functional group reactivity, contributing to their chemical stability^41^. Their structural robustness and thermal origin therefore increase the likelihood of preservation in archaeological contexts, where post-depositional alteration and degradation typically reduce the detectability of more fragile constituents. These properties make such chemomarkers particularly suitable for the molecular identification of cedar tars in ancient residues.

### Assessing the preservation of cedar tar chemomarkers in archaeological samples

Building on the identification of the cedar tar chemomarker set, we conducted a proof-of-concept analysis to evaluate their relevance in archaeological contexts. This assessment aimed to test whether the distinctive molecular fingerprint established for cedar tars can be reliably detected and interpreted in complex ancient organic residues. Accordingly, we analysed samples from ancient Egyptian mummification balms preserved in canopic jars from the Late Period (664–332 BCE; for further information on the archaeological materials see Supplementary Information), as previous analyses have demonstrated that embalming materials frequently contain cedar-derived substances mixed with other substances^21,22,24,60,61^.

**Fig. 5:**
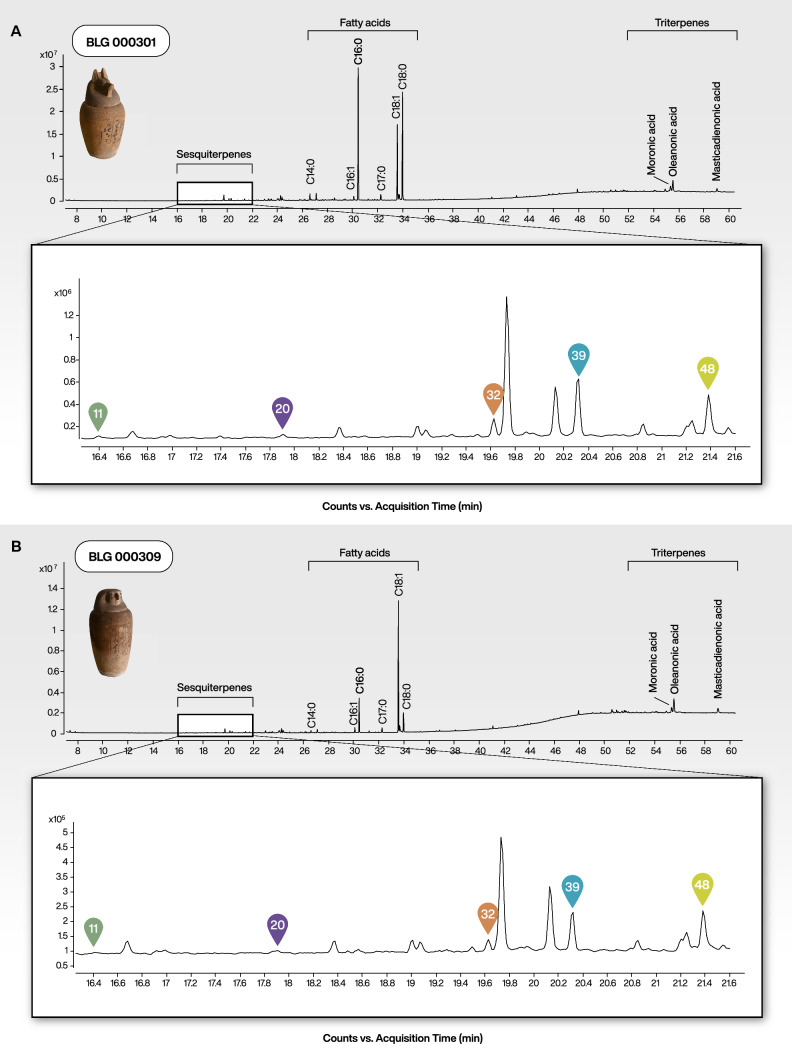
Total ion current (TIC) GC–MS chromatograms of archaeological samples BLG 000301 and BLG 000309 from ancient Egyptian canopic jars. (**A**) Chromatogram of sample BLG 000301, showing the overall distribution of fatty acids, sesquiterpenes, and triterpenes. The lower panel provides an expanded (zoomed-in) view, highlighting key features (#11, #20, #32, #39 and #48) characteristic of cedar tars. (**B**) Chromatogram of sample BLG 000309 with zoomed-in view showing tar chemomarkers. See Supplementary Fig. S5 for comparison of chromatograms with those obtained from modern cedar tar samples and Fig. S6 for a zoomed-in view of the triterpenoids.

The results of the analysis by GC–MS of samples from two canopic jars from the archaeological collection of the University of Jena (Sammlung Antiker Kleinkunst der Friedrich-Schiller-Universität Jena, Germany) revealed a complex molecular composition. Both samples (BLG 000301 and BLG 000309) showed a high similarity with the presence of common fatty acids, sesquiterpenes and triterpenes (Fig. 5). While the sesquiterpenes were present at lower relative abundance compared to fatty acids, we identified the primary chemomarkers of cedar tar: ar-himachalene (#39), dihydro-ar-turmerone (#48), cuparene (#32), dihydrocurcumene (#20) and 1-methyl-3,5-diisopropylbenzene (#11) (Fig. 5). Additionally, several triterpenoids (i.e. moronic, oleanonic, and masticadienonic acids), which are characteristic markers of *Pistacia* resin, were also detected^62^. Both samples were dominated by fatty acids, with major constituents including palmitic acid (C_16:0_), stearic acid (C_18:0_), and monounsaturated fatty acids, such as octadecenoic acid (C_18:1_) and hexadecenoic acid (C_16:1_), alongside with minor amounts of myristic acid (C_14:0_) and heptadecanoic acid (C_17:0_). These chemical distributions were typical of degraded lipid mixtures, such as plant fixed oils or mammalian fats. The relative intensity of unsaturated C_18:1_ differed between the two samples, being more pronounced in BLG 000309, which may reflect differences in the original lipid source or in degradation processes during burial. C_18:1_ and C_16:1_ occur in both vegetable oils and animal fats, so their presence alone did not identify the lipid source^63^. Heptadecanoic acid (C_17:0_) is sometimes taken as indicative of ruminant lipids; however, its low abundance here, together with the absence of the corresponding branched-chain isomers, more plausibly pointed to bacterial alteration of the lipid matrix^64^. Considering the overall pattern, the balms most likely contained a mixture of degraded animal fats and plant oils. Despite the mummification balms being a blend of organic substances, the detection of multiple chemomarkers associated specifically with cedar tars enabled the identification of *Cedrus*-derived tar as one of the components in the embalming materials, alongside *Pistacia* resin and plant oil/animal fats.

## Discussion

This study demonstrates that it is possible to distinguish cedar tar from other types of manufactured plant-derived materials, as well as unprocessed plant resins, even in archaeological contexts. By identifying a distinct set of chemomarkers, we established a molecular signature that reflects both the taxonomic origin of cedar and the thermal transformation induced by dry distillation. Having shown that these chemomarkers can be preserved in archaeological samples, we also review earlier studies to assess whether the same compounds had previously been detected, even if not interpreted in the context of cedar tar occurrence.

Indeed, several papers have reported the presence of these compounds in ancient residues, although their association with cedar tar was not recognized at the time^20,21,42,65–67^. For example, dihydro-ar-turmerone (#48) has been previously detected in a study investigating ancient Egyptian embalming substances from the Natural History Museum of the University of Florence, Italy^65^. This compound was found in four of the six analysed samples, and in two of them appeared alongside cuparene (#32) and calamenene (#36). These findings were not further discussed in the corresponding study, likely due to the complexity of these ancient samples, which also contained other markers associated with coniferous plants. The combination of dihydro-ar-turmerone (#48) and cuparene (#32) has also been observed in two samples of a Late Period mummy of a priest from the site of Sawa, Egypt^67^. In another study of embalming residues from the mummy Nestawedjat, all three chemomarkers—dihydro-ar-turmerone, cuparene, and ar-himachalene (#48, #32 and #39)—have been identified^66^. The authors have interpreted these compounds as indicative of “resinous plant species of the Pinaceae and Cupressaceae families”, but they have not proposed a more specific explanation such as the presence of cedar tar. Due to the presence of three of the chemomarkers highlighted in the present study, we suggest that this embalming substance might indeed contain cedar tar.

Sarret et al.^20^ have analysed a black residue from an Egyptian jar dating to the First or Second Dynasty (ca. 3100–2700 BCE) and have reported the presence of both himachalene-type sesquiterpenes and cuparenes. They have interpreted these findings as evidence for the use of two distinct botanical sources: *Cedrus libani*, based on himachalene sesquiterpenoids, and Cupressaceae (e.g., junipers or cypresses), based on cuparene sesquiterpenoids. While the co-occurrence of these compounds could reflect a mixture of materials, our findings now suggest that they may also originate from a single product, namely cedar tar. Similarly, Fulcher et al.^21^ have identified both compounds, ar-himachalene and cuparene (#39 and #32), in embalming materials from the 22nd Dynasty (ca. 945–715 BCE). While they have acknowledged the possibility that these compounds could derived from different plant inputs, they have also noted that their continuous co-occurrence across multiple samples, might point to a shared botanical origin.

The presence of these compounds in earlier datasets underscores the feasibility of detecting them in archaeological materials and highlights the value of re-evaluating legacy data through the lens of updated chemotaxonomic frameworks. Our findings help resolve long-standing ambiguities surrounding different cedar-derived products. By establishing a robust metabolomic framework, this study not only confirms the use of cedar tar in ancient embalming practices but also improves the interpretive resolution of chemically complex archaeological organic residues. These results emphasize the importance of revisiting historical datasets using refined sets of chemomarkers, thereby enabling more accurate interpretations of material compositions and offering deeper insights into ancient technologies and material practices.

While the presence of specific chemomarkers can serve as a strong indicator of cedar tar, interpreting their absence requires greater caution. It is a well-established principle in archaeological science that the absence of evidence is not necessarily evidence of absence. However, in the present study, the chemomarkers used are characterised by their relative thermal and chemical stability. Therefore, their complete absence, particularly when more labile or volatile constituents are still detectable, suggests that cedar tar was likely not originally present. Nevertheless, the possibility of partial loss through microbial, chemical or enzymatic degradation over time cannot be entirely excluded.

Despite the potential of this study, several limiting factors must be acknowledged. In particular, the geographical origin and production context of modern reference materials may influence the detailed chemical composition of cedar-derived products. At present, however, there is insufficient archaeological and textual evidence to reconstruct in detail how cedar tar was produced in ancient Egypt. Accordingly, the modern cedar tar samples used here served as reference materials for dry-distilled cedar tar as a product category, rather than as direct technological or regional analogues.

Future work should address these limitations by expanding comparative metabolomic approaches across a wider taxonomic and technological range and larger sample sizes. Controlled experimental studies replicating ancient production processes and degradation experiments will help to identify transformation products with greater specificity. Applying such a metabolomic framework systematically to archaeological contexts beyond Egyptian embalming will clarify the broader roles of cedar tar in medicine, ritual, and craft technologies.

Lastly, this study demonstrates the potential of metabolomics in archaeological science. Rather than representing a fundamentally new analytical technique, the metabolomics approach applied here builds on long-established GC–MS-based archaeochemical methods by providing a structured framework for systematically exploring the molecular complexity of plant materials. Archaeo-metabolomics provides new opportunities for investigating ancient plants, by combining comprehensive feature detection, feature alignment, systematic comparison of metabolite profiles across samples, with multivariate statistical analysis. This approach enables the identification of subtle yet diagnostic molecular signatures even in degraded and chemically complex mixtures. By introducing new chemomarkers to distinguish closely related plant-derived products, our study improves the molecular identification of cedar tar. More broadly, this work contributes to tracing the long history of human technological innovation that began when Neanderthals first mastered tar production. These results open new avenues for reconstructing past technologies, human–environment interactions, and cultural practices.

## Materials and methods

### Materials

Methanol (MeOH), dichloromethane (DCM) used for the analyses were obtained from Sigma-Aldrich (Munich, Germany), as well as the analytical standard (+)-longifolene. 1-(4-methylphenyl)ethanone was purchased from ThermoFisher Scientific (Kandel, Germany), and (+)-(*S*)-ar-turmerone from ABCR (Karlsruhe, Germany). Furthermore, oleanonic acid was obtained from Santa Cruz Biotechnology (Heidelberg, Germany), 7-oxodehydroabietic acid from Campro Scientific (Berlin, Germany), moronic acid from TCI chemicals (Eschborn, Germany), and dehydroabietic acid from Carbosynth (Berkshire, UK).

### Sample collection of modern botanical samples of *Cedrus* products

Modern botanical reference samples of *C. atlantica* resin were collected in March 2021 from the arboretum of Pierrefeu-du-Var (France). *C. atlantica* tar samples were obtained in October 2009 from Les Arômes de Ben Sim (resp. M. El Hachimi) in Azrou (Morocco). The distillates were traditionally produced using the *per ascensum* process^68^, a dry distillation process in which volatile products migrate upward during heating. *C. atlantica* essential oils have been obtained from Florihana Distillerie (Caussols, France). Supplementary Table S1 provides the list of the reference samples investigated. All plant materials (here: *C. atlantica* resins) were collected and used in accordance with relevant institutional, national, and international guidelines and legislation. The study complies with the IUCN Policy Statement on Research Involving Species at Risk of Extinction and the Convention on International Trade in Endangered Species of Wild Fauna and Flora (CITES).

### Sampling of ancient mummification balms

Samples (approx. 50 mg) were taken from two canopic jars (inventory nos. FSU 106 and 107) housed in the archaeological collection of the Friedrich Schiller University Jena (Germany). The jars were covered with lids in the form of animal heads and come from ancient Egyptian funerary contexts. Further details on the dating, archaeological provenance, collection are provided in the Supplementary Information. The contents within the canopic jars consisted of a black, dense, and compact material partially deposited at the bottom and adhering to the inner walls of the jars. One sample from each jar was collected from the bottom deposit. Prior to sampling, approximately 1 mm of the surface layer was carefully removed using clean disposable scalpels to eliminate potential contaminants. Material was then taken from the underlying layer and transferred into pre-combusted (500 °C, 8 h) glass vials for storage until further processing.

### Sample preparation for GC–MS analysis

Since tars and essential oil samples are fully soluble in DCM, no additional extraction steps were required. For the preparation of essential oils, 20 μL of essential oil was placed in 1.5 mL glass vials. Each sample was then dissolved in 980 μL of DCM and briefly (~ 20 s) mixed by vortexing to ensure complete dissolution. Tar pieces were dissolved in 1 mL of DCM and thoroughly mixed afterwards. Aliquots (100 μL) of the samples have been transferred to vials for GC–MS analysis. The resin samples were finely homogenized using a mortar and pestle to ensure uniform particle size. Solvent extraction was performed utilizing 20 mg of resin powder and 1 mL of a DCM MeOH mixture (2:1, v/v). The samples were subjected to 15 min of ultrasonication to enhance analyte dissolution, followed by 10 min of centrifugation. The supernatants were transferred to new vials for GC–MS analysis.

The extraction of archaeological samples followed established protocols^69,70^. Briefly, a mixture of DCM/MeOH (2:1, v/v) was used for analyte extraction (50 mg of sample and 2 mL solvent mixture), followed by 15 min of ultrasonication and 10 min centrifugation, repeated three times to increase the extract concentration. Aliquots of the resulting extracts of the archaeological samples were derivatized with *N*,*O*-bis(trimethylsilyl)trifluoroacetamide (BSTFA) containing 1% TMCS and 4 μL of pyridine at 70 °C for 1 h, in order to increase the volatility and chromatographic detectability of polar compounds, particularly fatty acids commonly present in ancient balm residues.

### GC–MS analysis

GC–MS analysis was conducted using an Agilent 8890 GC system with a 5977B single-quadrupole mass selective detector (Agilent Technologies). Compound separation was achieved on a 60 m × 250 µm HP-5 ms capillary column (Agilent) with a 0.25 µm film thickness. The system operated in electron impact (EI) ionization mode at 70 eV, with helium serving as the carrier gas at a constant flow rate of 1.2 mL/min. The GC oven temperature program for analysing modern cedar and pine samples was set at 50 °C, held isothermally for 1 min, then ramped to 150 °C at a rate of 30 °C/min (1 min hold), increased again at 5 °C/min to 200 °C (1 min hold). The temperature was then ramped to 320 °C at a rate 15 °C/min with final hold for 1 min. The total run time was 34 min with a solvent delay of 7 min. Injection volume was 1 μL with a split ratio of 5:1. The scanning range for the temperature program was set from *m/z* 40 to 600 amu. Samples were analysed in triplicates with blanks in between. In order to improve the separation and detection of late-eluting high-molecular-weight compounds, particularly triterpenoids, an additional GC temperature program was applied to the trimethylsilylated archaeological samples. This program initiated at 50 °C (held for 2 min), followed by a ramp to 120 °C at 30 °C/min (2 min hold), and then a second increase to 320 °C at 5 °C/min with a final hold of 15 min. 1 μL of each sample was injected with a split ratio of 10:1. The scanning range was set from *m/z* 40 to 700 amu.

### Quality control and quality assurance (QC/QA)

Quality control (QC) and quality assurance (QA) measures were implemented throughout the analytical and data-processing workflow to ensure data quality, reproducibility, and robustness of the GC–MS-based metabolomics analyses. At the pre-analytical stage, extensive measures were taken to minimize contamination. Archaeological samples were prepared in a dedicated clean laboratory reserved for ancient materials, while modern reference samples used for chemomarker identification were prepared in a separate laboratory to avoid cross-contamination. Glassware was pre-combusted where appropriate, and disposable laboratory consumables were used throughout sample preparation. At the analytical stage, modern reference samples were analysed in triplicates to assess intra-sample variability. All modern reference samples were analysed within a single uninterrupted GC–MS sequence, thereby minimizing potential inter-run variability. Samples were injected in a randomized order and blanks were included after every sample throughout the analytical sequence to monitor background contamination, and potential carryover. GC–MS raw data were processed using a consistent workflow in MZmine (see below). Features detection, filtering, deconvolution, isotope filtering, alignment, and gap filling were performed using predefined and stringent parameters to retain only reproducible and chemically meaningful features. Prior to multivariate statistical analysis, the data matrix was normalized by the sum of chromatographic peak areas, log_10_-transformed, and mean-centered to reduce injection-to-injection variability and enable comparison of relative metabolite abundances across samples.

### Metabolomics data processing, statistical analyses and chemomarker annotation

GC–MS raw data were acquired using Agilent MassHunter software (Version 10.0) and converted to the open mzML format using MSConvert (Version 3.0; Proteowizard). The exported mzML files were processed using MZmine (Version 4.6.1) following an optimized workflow for clean and reproducible feature detection in complex organic matrices. Centroided mass spectra were used for downstream analysis. Initial mass detection was performed with a noise threshold of 1.0E02, ensuring the capture of low-abundance yet chemically relevant features. Chromatograms were constructed using the “Chromatogram Builder” function with a minimum of 7 consecutive scans, a minimum intensity threshold of 1.0E03, a minimum absolute height of 1.0E02 and a “scan-to-scan *m/z* tolerance” of 0.5 Da. Peak deconvolution was achieved using the “local minimum resolver”, applying an 80% chromatographic threshold, a minimum relative height of 2%, and a minimum absolute peak height of 2.0E02. Peaks were retained only if they spanned at least 3 data points and met a minimum top-to-edge intensity ratio of 1.7. To further isolate true compound signals, GC-EI spectral deconvolution was performed using a retention time tolerance of 0.03 min, a minimum of 8 fragment signals per pseudo-spectrum and a minimum shape similarity of 0.70. ^13^C isotope grouping was applied with an *m/z* tolerance of 0.03 Da and a retention time tolerance of 0.5 min, ensuring isotopologues were correctly grouped without removing MS/MS-linked features.

Feature alignment across samples was conducted using the “Join Aligner” function, with a mass tolerance of 0.3 Da, a retention time tolerance of 0.15 min, and weighting factors of 2 and 1 for *m/z* and retention time, respectively. Gap filling was performed using an *m/z* tolerance of 0.2 Da, a retention time tolerance of 0.10 min, an intensity tolerance of 0.1%, and requiring a minimum of 2 scans per feature. The resulting data matrix (Supplementary Data 1) was exported as a CSV file for statistical analysis (PCA, PLS-DA) and chemical marker annotation.

Statistical analysis of the resulting data matrix was carried out using the online platform MetaboAnalyst 6.0. The resulting data matrix was normalized by the sum of chromatographic peak areas, log-transformed and mean-centered as described in Paix et al.^71^. Statistical analyses were performed to explore compositional differences between the cedar-derived products. Principal component analysis (PCA) was used to visualize the variance structure in the dataset, followed by partial least squares discriminant analysis (PLS-DA) to identify discriminating features among product types. Discriminant variables (features) were selected based on their statistical significance and variable importance in projection (VIP) scores, which informed the prioritization of features for chemical annotation. To further assess differential abundance, a volcano plot was generated, combining fold-change and *p* value thresholds to highlight metabolites that were both substantially enriched and statistically significant in cedar tar relative to other cedar products. Annotation of discriminant features was performed by comparing *m/z* values, retention times, and mass spectra with those in the NIST EI spectral library and using commercial analytical standards where available.

## Data and materials availability

All data are available in the main text, the supplementary material and on a data repository (10.6084/m9.figshare.30665321). 

## Supplementary Information

Supplementary Information 1.

Supplementary Information 2.
