# Supplementary material for: An archaeo-metabolomics approach for identifying cedar tar in archaeological samples: differentiating plant products and production processes
Source: Sci Rep. 2026 May 5;16:14280. doi: 10.1038/s41598-026-50080-6 (PMC13144440; doi:10.1038/s41598-026-50080-6)
Supplement: Supplementary file 1 — Supplementary Information 1. [file 41598_2026_50080_MOESM1_ESM.pdf]

Supplementary Materials for

An archaeo-metabolomics approach for identifying cedar tar in archaeological samples: Differentiating plant products and production processes

Barbara Huber* *et al.*

*Corresponding author. Email: huber@gea.mpg.de

This PDF file includes:

Supplementary Text
Figs. S1 to S6
Tables S1

Other Supplementary Materials for this manuscript include the following:
Data S1

Supplementary Text

<u>Historical context of the ancient Egyptian canopic jars</u>

Samples for archaeometabolomics analysis were taken from two canopic jars held in the former archaeological collection of Jena University. This collection was founded in 1846 by the philologist Carl Wilhelm Goettling (1793–1869) and was originally housed in the former city palace. Supported by the newly established Archaeological Society, the collection expanded rapidly through acquisitions and donations. By the early 20th century, under Ferdinand Noack and Botho Graef, it comprised more than 600 plaster casts of ancient sculptures and over 1,700 original artifacts, primarily ceramics, terracottas, bronzes, and marble objects from the wider Mediterranean world, dating from the Bronze Age to Late Antiquity. Only a small number of Egyptian objects are held in the collection, many of them originating from private donations, including the canopic jars under study. They were donated by the merchant Friedrich Batsch (1789–1834), but the precise provenance of the pieces is not known. The first museum catalogue of the collection notes simply that Batsch brought the Egyptian pieces (Aegyptiaca) from Egypt in 1816, and after his death they were donated to the museum by his widow and a Major Batsch (*1*).

The two canopic jars examined in this study date to the Late Period (c. 664–525 BCE). One jar bears a lid in the form of a jackal head (Fig. S1), while the other has a falcon-head lid (Fig. S2). Canopic jars held the internal organs removed during mummification, with each vessel associated with one of the Four Sons of Horus, who protected the organs for the afterlife. Duamutef, depicted as a jackal, safeguarded the stomach, whereas Qebehsenuef, represented with a falcon head, protected the intestines. In both jars, the organs had been removed and only residues of mummification balms remained, deposited on the inner walls and the base of the vessels. Both jars are inscribed with protective formulae invoking Osiris and referring to the deceased, *Hr-Udsha* (*2*).

References:

1 Goettling, Carl Wilhelm: Verzeichniss der Gegenstände des im Jahr 1846 gegründeten archäologischen Museums der Universität Jena (Jena 1846) p. 6.

2 Graen, Dennis (ed.): Ägypten. Unbekannte Schätze aus Thüringer Sammlungen, Ausstellungskatalog, Beiträge aus den Sammlungen der Universität Jena, vol. 1 (Jena 2012) p. 44-45.

Fig. S1.

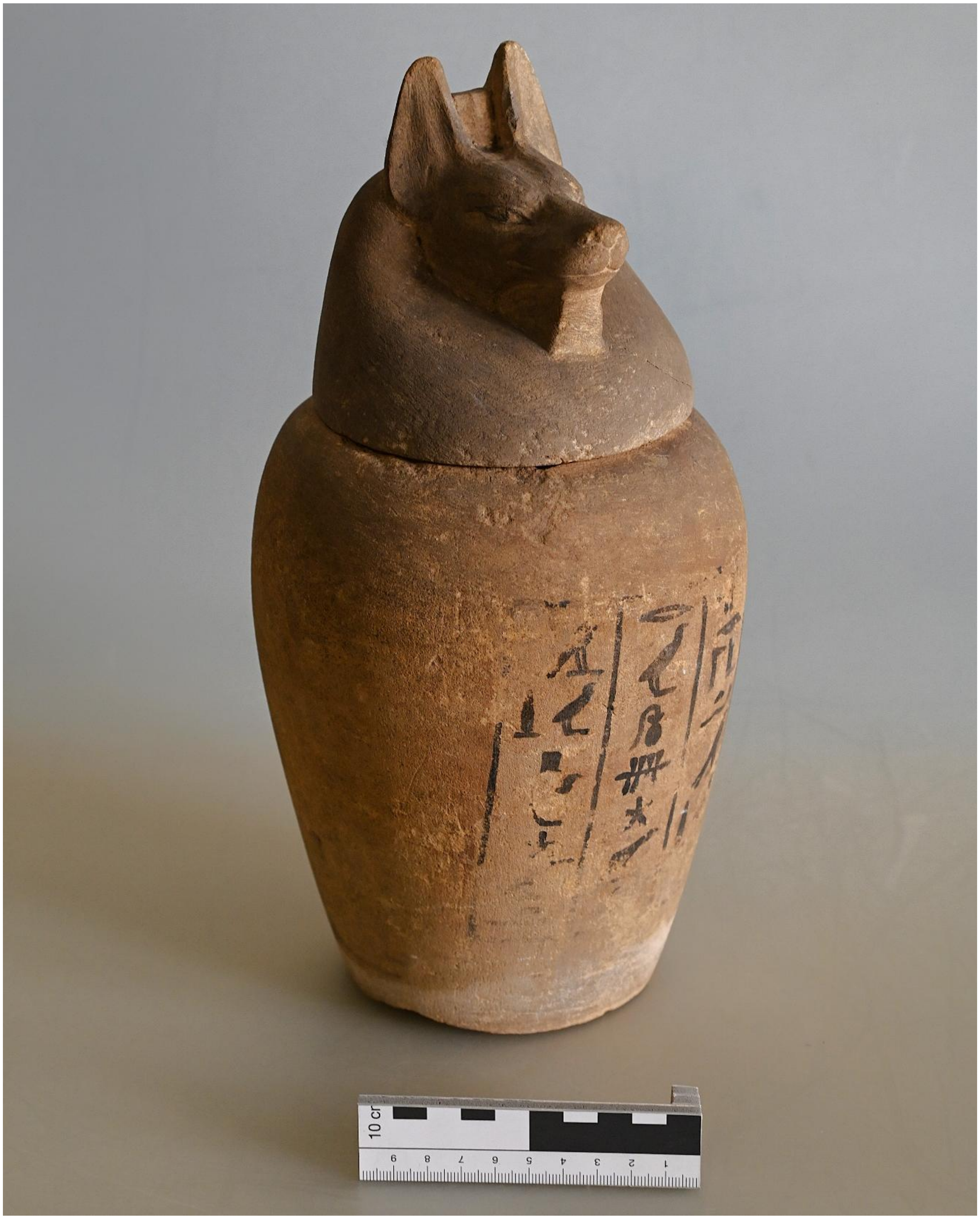

Canopic Jar with Jackal-Head Lid. Friedrich-Schiller-Universität Jena, Hilprecht Collection / Collection of Classical Antiquities, Inv. no. 106, Late Period (664–525 BCE); Height of jar: 25.1 cm.

Fig. S2.

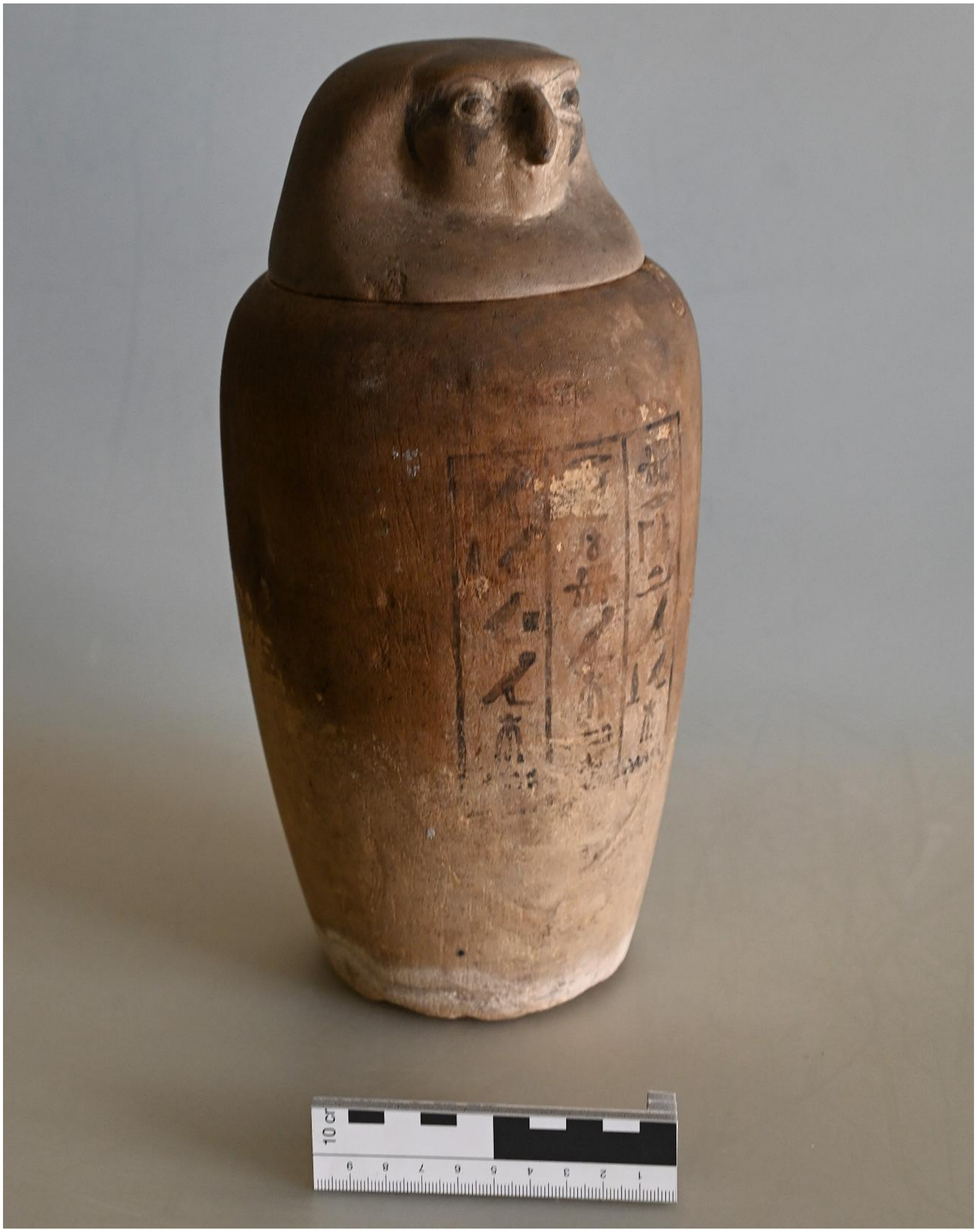

Canopic Jar with Falcon-Head Lid. Friedrich-Schiller-Universität Jena, Hilprecht Collection / Collection of Classical Antiquities, Inv. no. 106, Late Period (664–525 BCE); Height of jar: 31.4 cm.

Fig. S3.

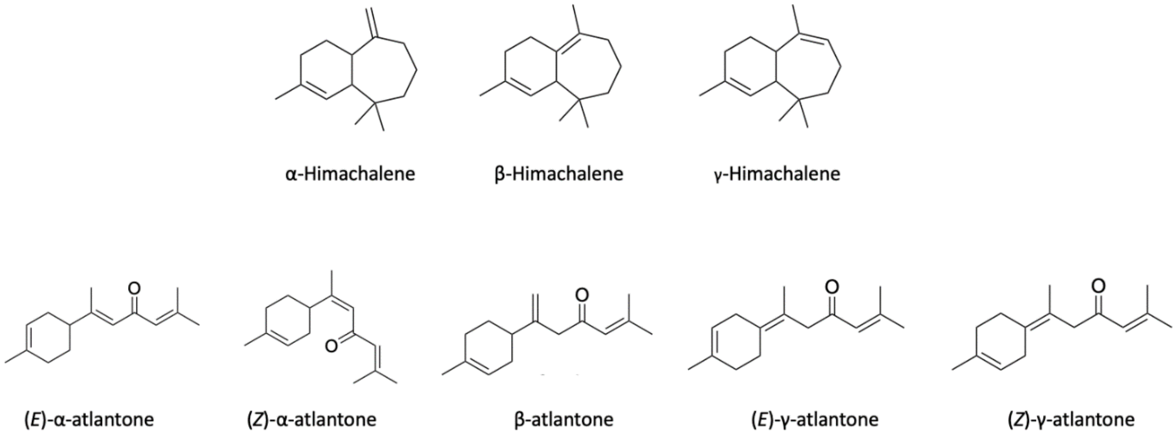

Chemical structures of major sesquiterpenoid compounds **in** *Cedrus* sp**p**. Top row: himachalene isomers (α-, β-, and γ-himachalene)**.** Bottom row: atlantone derivatives, including (*E*)-α-atlantone, (*Z*)-α-atlantone, β-atlantone, (*E*)-γ-atlantone, and (*Z*)-γ-atlantone**.**

Fig. S4

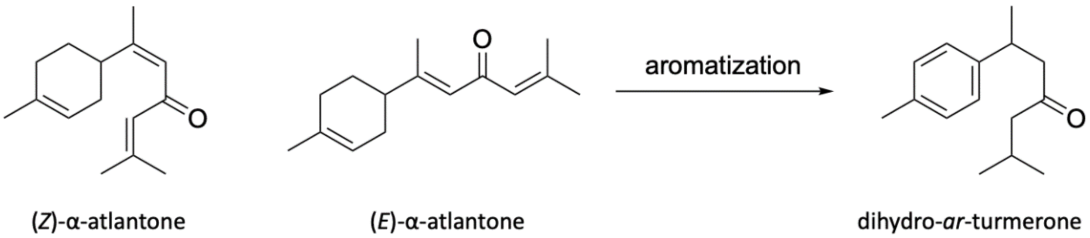

Proposed formation pathway of dihydro-*ar*-turmerone (#48) from (*Z*)- and (*E*)-α-atlantone via aromatisation. Both geometric isomers of α-atlantone can undergo double-bond rearrangement in the presence of a catalyst and heat.

Fig. S5.

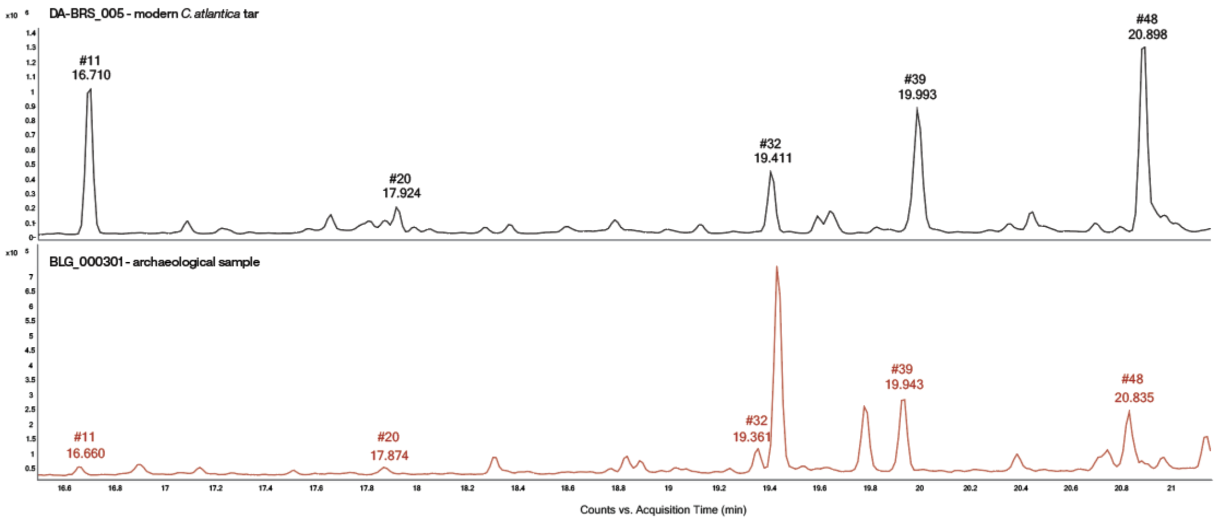

Comparison of partial GC-MS total ion chromatograms (TIC) of modern *Cedrus atlantica* tar reference sample (DA-BRS_005; top) and an archaeological sample (BLG_000301; bottom). The same set of compounds is detected in both samples, although a slight retention time shift between chromatograms is observed.

Fig. S6

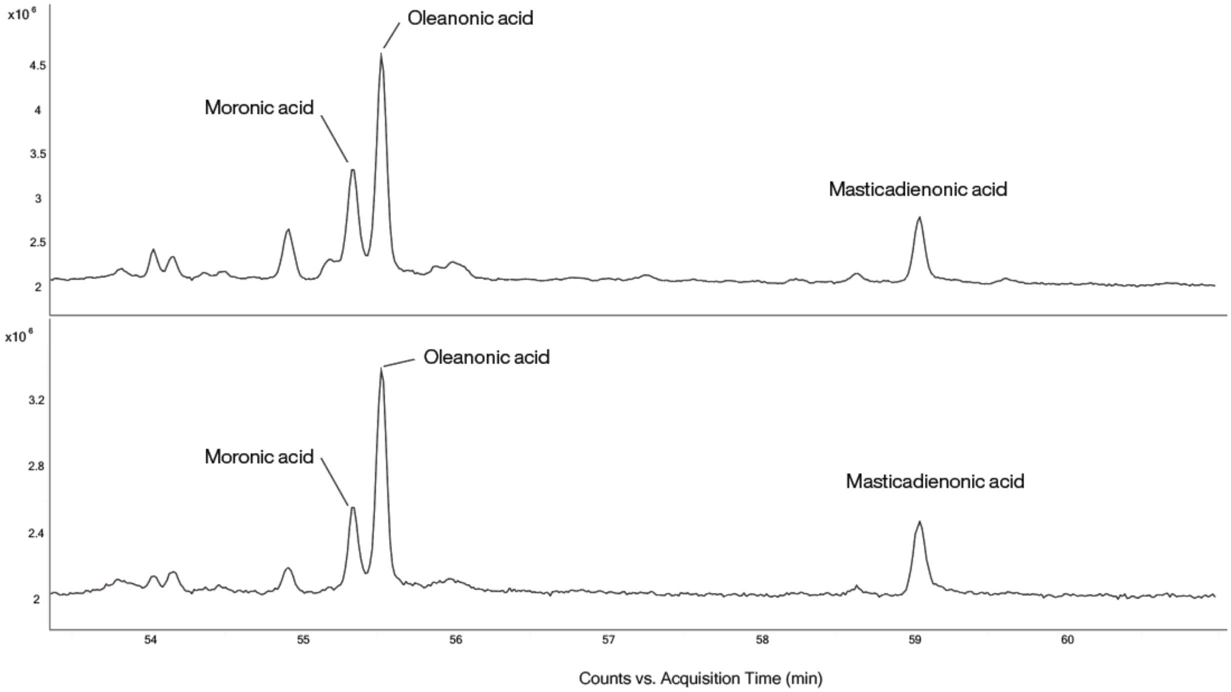

Expanded GC-MS chromatograms showing the triterpenoid fraction detected in archaeological samples BLG 000301 (upper) and BLG 000309 (lower) from ancient Egyptian canopic jars. The chromatograms highlight characteristic triterpenoid compounds including moronic acid, oleanonic acid, and masticadienonic acid. These compounds are indicative of Pistacia-derived resins present in the embalming mixtures.

Table S1.

**DA code**	**Lab no. Nice**	**Product**	**Species**	**Provenance**
DA-BRS_001	MR2041	Cedar tar	*Cedrus atlantica*	Morocco
DA-BRS_002	MR2046	Cedar tar	*Cedrus atlantica*	Morocco
DA-BRS_003	MR2047	Cedar tar	*Cedrus atlantica*	Morocco
DA-BRS_005	MR2049	Cedar tar	*Cedrus atlantica*	Morocco
DA-BRS_006		Cedar essential Oil	*Cedrus atlantica*	France
DA-BRS_007		Cedar resin	*Cedrus atlantica*	France

Overview of modern reference samples of cedar products analyzed in this study.

Data S1. (separate file)

Feature matrix of cedar products showing detected peaks (rows) across samples (columns). Cell values indicate peak intensity after preprocessing (peak detection, deconvolution, alignment).
